# Near-Infrared Spectroscopy measurements are reliable for studying patellar bone hemodynamics and affected by venous occlusion, but not by skin compression

**DOI:** 10.1186/s40634-023-00709-6

**Published:** 2023-11-29

**Authors:** Martin J. Ophey, Anne Westerweel, Maxime van Oort, Robert van den Berg, Gino M. M. J. Kerkhoffs, Igor J. R. Tak

**Affiliations:** 1 IJsveldFysio – Private Physical Therapy Clinic, Nijmegen, The Netherlands; 2 Department of Orthopaedic Surgery and Sports Medicine, Amsterdam UMC Location University of Amsterdam, Meibergdreef 9, 1105 AZ Amsterdam, The Netherlands; 3 ESP Science and Education, Vienna, Austria; 4 RU - Radboud University, Biomedical Sciences, Nijmegen, The Netherlands; 5 FH Burgenland, Physical Therapy Department, University of Applied Science, Pinkafeld, Austria; 6 AIM - Austrian Institute of Management, Advanced Physiotherapy & Management, Eisenstadt, Austria; 7 Amsterdam Collaboration On Health & Safety in Sports (ACHSS), IOC Research Center, Amsterdam, The Netherlands; 8 Academic Center for Evidence-Based Sports Medicine (ACES), Amsterdam, The Netherlands; 9 Physiotherapy Utrecht Oost – Sports Rehabilitation and Manual Therapy, Utrecht, The Netherlands

**Keywords:** Patellofemoral pain, Reliability, Near-Infrared Spectroscopy

## Abstract

**Purpose:**

According to the homeostasis model, patellofemoral pain (PFP) results from disturbed homeostasis due to vascular insufficiency in the anterior knee. Near-Infrared Spectroscopy (NIRS) measures relative changes in concentrations (in µmol/cm^2^) of (de-)oxygenated hemoglobine (HHb and O_2_Hb). The aims were to: 1) investigate the characteristics of the NIRS signal derived from the patella during experiments affecting hemodynamics in healthy controls, and 2) determine the test–retest reliability of NIRS in positions clinically relevant for PFP patients.

**Methods:**

Two experiments were conducted on 10 healthy controls and analysed using Student’s *t*-test. Reliability (*ICC*_*2,1*_) was evaluated for two activities (‘Prolonged Sitting’ and ‘Stair Descent’) in five PFP patients and 15 healthy controls, performed twice within five days.

**Results:**

The NIRS signal (HHb and O_2_Hb) showed a statistically significant increase (*p* < .001 – .002) on all optodes (30, 35, 40 mm) during ‘Venous Occlusion’ (*M* = 1.0 – 2.0), while it showed no statistically significant change (*p* = .075 – .61) during ‘Skin Compression’ (*M* = -0.9 – 0.9) on the 30 and 35 mm optode. Reliability of NIRS (HHb and O_2_Hb) ranged from moderate to almost perfect (*ICC*_*2,1*_ = .47 – .95) on the 30 mm optode for ‘Prolonged Sitting’, and from moderate to substantial (*ICC*_*2,1*_ = .50 – .68) on the 35 mm optode for ‘Stair Descent’.

**Conclusions:**

Patella NIRS measurements are affected by venous occlusion, but not by skin compression, and are sufficiently reliable as research application to compare real-time patellar bone hemodynamics. These insights may assist to improve effectiveness of evidence-based treatment strategies for PFP.

**Trial registration:**

ISRCTN Trial Registration under number: 90377123.

## Background

Patellofemoral pain (PFP) is a common and difficult to treat clinical condition. The annual prevalence in the general population ranges from 16 to 29% [48]. Despite evidence-based treatment recommendations advocating multimodal interventions, including exercise therapy, more than 40% of PFP patients reportedly continue experiencing knee symptoms five to eight years after diagnosis [8, 24, 27, 29, 30].

Since the 1980s, patellofemoral maltracking (PFM) has been the most widely accepted model explaining the genesis of PFP [39]. This model refers to altered patellofemoral kinematics and contact forces, as it may be the result of impaired quadriceps function or lateral soft tissue tightness [29, 45]. While some studies have expressed serious methodological concerns about the validity of the PFM model [20, 27], the aforementioned multimodal treatment recommendations are all based on this model. This dependence might account for the poor long-term treatment outcomes.

According to the more recent homeostasis model [16, 39], PFP is the result of a disturbed homeostasis of structures of the anterior part of the knee, including soft and osseous tissues, due to supraphysiologic loading [39]. Although no data from larger prospective trials conclusively permit causal inferences on the validity of this model, evidence aligning with the homeostasis model consists of morphologic changes of the retinacula [26, 45], including neovascularisation and hyperinnervation [40, 41], and increased intraosseous water content and pressure [22, 33]. Some authors suggest that these changes result from hypoxia due to vascular insufficiency of the peripatellar anastomotic ring [19, 40, 42, 47]. Only a few cross-sectional studies have examined patellar blood flow in PFP patients, suggesting reduced drainage time of the venous system, reduced pulsatile blood flow, and statistically non-significant differences in blood perfusion measured with intraosseous phlebography, photoplethysmography, and dynamic contrast-enhanced magnetic resonance imaging (MRI), respectively [2, 36, 51].

Near-Infrared Spectroscopy (NIRS) is an optical, non-invasive method using a light-source and -detector that enables continuous assessment of bone hemodynamics [5, 32]. While NIRS is an established tool in muscle physiology and transcranial cerebral circulation [43, 52], it has not been used to evaluate homeostasis of the patella in PFP patients.

Before studying differences in hemodynamics between PFP patients and healthy controls, the current study aimed to: 1) investigate the characteristics of the NIRS signal derived from the patella during interventions affecting hemodynamics of the anterior knee in healthy controls, and 2) determine the test–retest reliability of NIRS derived from the patella in positions clinically relevant for PFP patients.

## Methods

### Participants

This observational study (level of evidence III) complied with the requirements of the Declaration of Helsinki [54]. The study protocol obtained approval by the Medical Research Ethics Committee of Amsterdam UMC location University of Amsterdam under number NL77408.018.21 and has been registered at the ISRCTN registry under number 90377123 prior to the start of the data collection.

A convenient sample was obtained by recruiting subjects from 1) a private physical therapy clinic, 2) the physical therapy education program of the HAN University of Applied Sciences and 3) the Radboud University in Nijmegen, the Netherlands. Recruitment took place in February and March 2022. A senior physical therapist (MO) screened subjects for eligibility through history taking and standardised physical examination. Criteria for inclusion and exclusion are detailed in Table 1.

**Table 1:** Criteria for Inclusion and Exclusion

	**Inclusion**	**Exclusion**
General	• Age: 18 to 40 years • Informed consent	• Previous or current clinical diagnosis of serious pathology (such as malignancy) • Previous or current other clinical diagnosis of specific knee conditions (such as patellar instability or dislocation, jumpers knee, meniscus tears, or other ligament injury) • Previous surgery (ankle, knee, hip, or lower back)
PFP patients	• Pain: ∘ experienced around and/or behind the patella ∘ aggravated by one or more of the following activities: squatting, stair ambulation, jogging/running, hopping/ jumping ∘ lasting for three months or longer ∘ not as a result of trauma • Experience worst pain levels of at least 3 / 10 on a visual analogue scale (VAS-W) during previous week	• Positive FABER/FADDIR (referred pain from the hip joint)
Healthy controls		• Previous diagnosis of PFP • Complaints of ankle, knee, hip, or lower back over the past six months requiring attention from a health care professional (physician, physical therapist), or resulting in missing more than one game, competition, or training

After inclusion, participants´ demographic data including gender, age, body mass index (BMI), current smoking status, and hours of sports per week were collected. Blood pressure was measured using an automatic blood pressure monitor (Omron M6; Omron Healthcare, Kyoto, JP) [1]. Additionally, all participants completed the Tegner score and the Anterior Knee Pain Scale (AKPS) [25, 49]. The Tegner score assesses the current physical activity level on a scale from 0 to 10 [9, 23], with higher scores indicating higher activity levels. The Dutch version of the Tegner score is reliable (*ICC* = 0.93 – 0.97) and valid with an internal consistency of *r* = 0.73 – 0.83 [17]. The AKPS, a 13-item questionnaire, measures subjective symptoms and functional disabilities associated with PFP. Higher scores correspond to fewer symptoms and disabilities [11], and the Dutch version is reliable (*ICC* = 0.98) and valid with an internal consistency of *r* = 0.78 – 0.80 [50]. Participants were instructed not to participate in any sports activity 12 h before the NIRS measurement.

### Near-Infrared Spectroscopy (NIRS)

Near-infrared light penetrates human tissue superficially with reported penetration depths up to 4 cm [4]. The NIRS device used was the PortaLite (Artinis Medical Systems; Elst, The Netherlands). The PortaLite is a continuous-wave device and the sensor, with a surface of 13.4 cm^2^, consists of three light emitting diodes arranged at 30, 35 and 40 mm distances from the detector. The diodes transmit near-infrared light at wavelengths of 760 and 820 nm, which have specific absorption characteristics for deoxygenated (HHb) and oxygenated hemoglobine (O_2_Hb). Given the measurement depth of NIRS (approximately half the sensor-detector distance [3]), this device measures HHb and O_2_Hb concentrations between 15 and 20 mm tissue depth. Superficial tissue (e.g., skin) has a non-significant confounding contribution on NIRS measurements if the superficial layer is less than 4 mm [18].

Evaluation of HHb and O_2_Hb concentrations using NIRS relies on the Beer-Lambert law modified with the ‘differential pathlength factor’ (DPF) [3, 15], which considers measurements taken in biological tissue. As no research with NIRS was conducted to examine patellar hemodynamics, the DPF was estimated using the equation: $\frac{1}{2}{\left(\frac{3{{\mu }{\prime}}_{s}\left(\lambda \right)}{{\mu }_{a}\left(\lambda \right)}\right)}^{1/2},$ which takes the absorption (μ_a_) and reduced scattering (μ_s_) coefficients of the human skull into account [44], resulting in a DPF of 8.66.

Data were sampled at a frequency of 10 Hz. NIRS measures relative changes of HHb and O_2_Hb concentrations, not absolute concentrations, compared to a baseline. Therefore, all measurements involved a 3-min baseline, and relative changes in HHb and O_2_Hb between baseline and experimental measurements (Δ) were calculated in µmol/cm^2^ [3]. The laboratory room maintained constant light, with a room temperature between 21 and 23℃.

### Patella skinfold and width

**Fig. 1:**
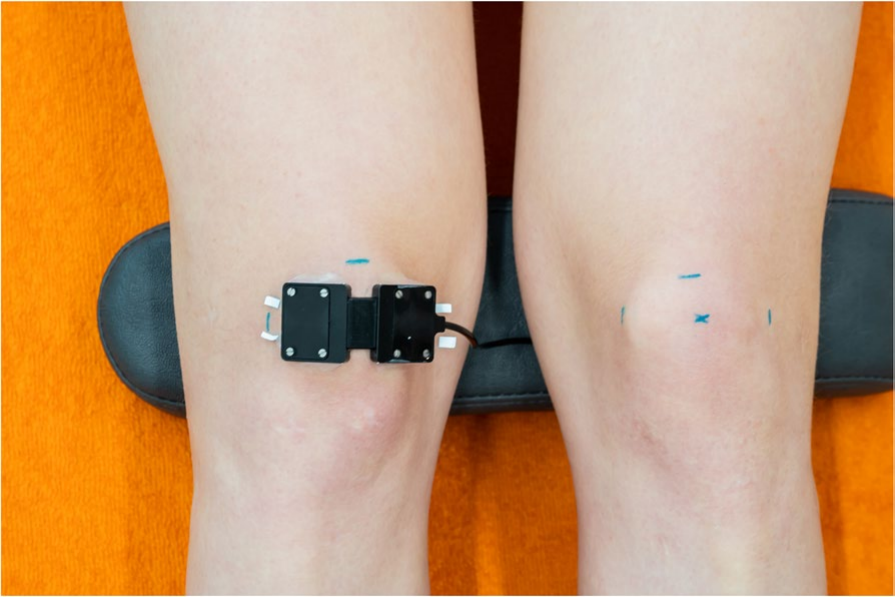
Marks on the skin of the left knee and sensor placement on the right knee

**Fig. 2:**
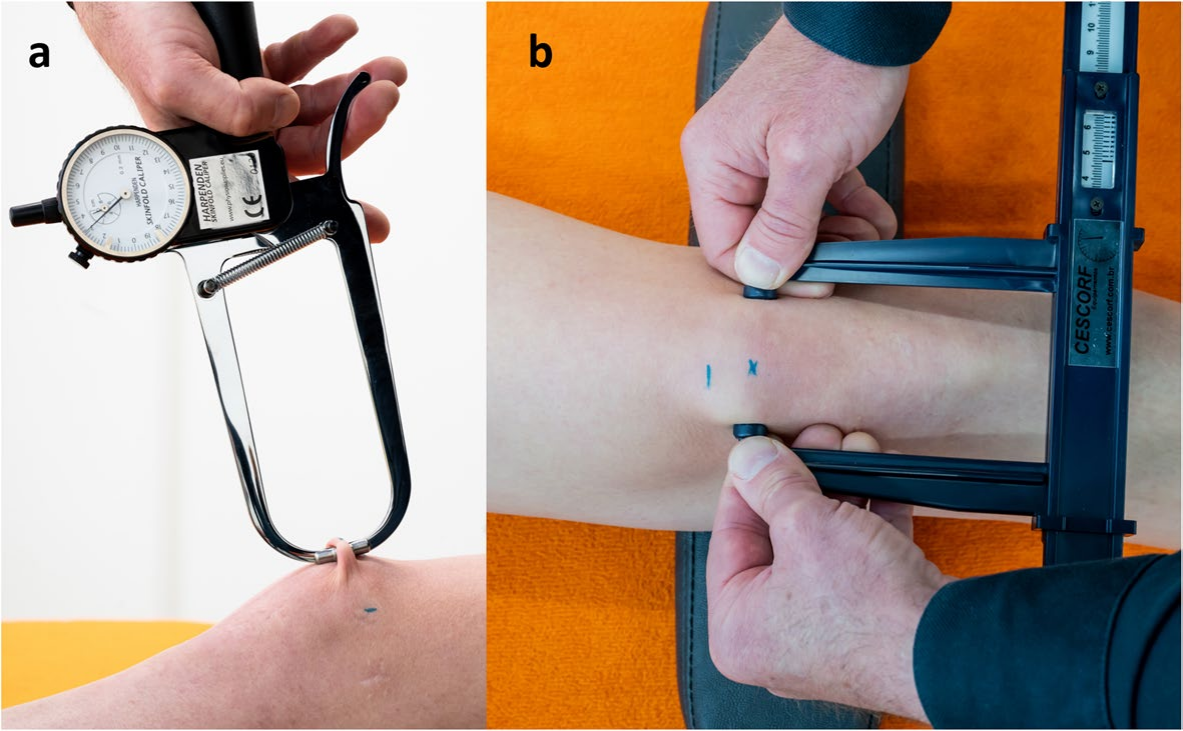
**a** Skinfold measurement and 2b Patella width measurement

For detailed anthropometric characteristics of the anterior knee, the prepatellar skinfold thickness and the patella width were measured. Marks were made with a skin marker (Edding 8020; Edding GmbH, Ahrensburg, GER) on the medial and lateral side of the widest part of the patella and a cross midway between these two marks. Additionally, the proximal edge of the patella was marked to avoid placing the sensor too proximal (Fig. 1, left knee), following guidelines from the International Society for the Advancement of Kinanthropometry (ISAK) [12]. After training by a certified and ISAK (level 1) registered dietitian, the skinfold thickness was measured (AW) at the cross at the center of the patella with a skinfold caliper (Harpenden; Baty International, West Sussex, UK) and the patella width at the widest part with a slide caliper (Innovare; Cescorf, Porto Alegre, BR) (Fig. 2a and b).

Both measurements were conducted twice, with a third measurement obtained if the difference between the repeated measurements exceeded 5%. The arithmetic mean was calculated for all measurements. Subsequently, the NIRS sensor was affixed to the skin using medical transparent double-sided adhesive tape (type 2181; 3 M, St. Paul, MN, US) with its middle on the cross, the three light sources positioned laterally and the detector medially (Fig. 1, right knee). An opaque cloth covered the NIRS sensor to minimise ambient light influence on the signal.

### Experiments

To investigate the characteristics of the NIRS signal derived from the patella, two experiments affecting hemodynamics of the anterior knee were conducted on the dominant leg of 10 healthy controls.

#### Experiment 1 ‘Venous Occlusion’

**Fig. 3:**
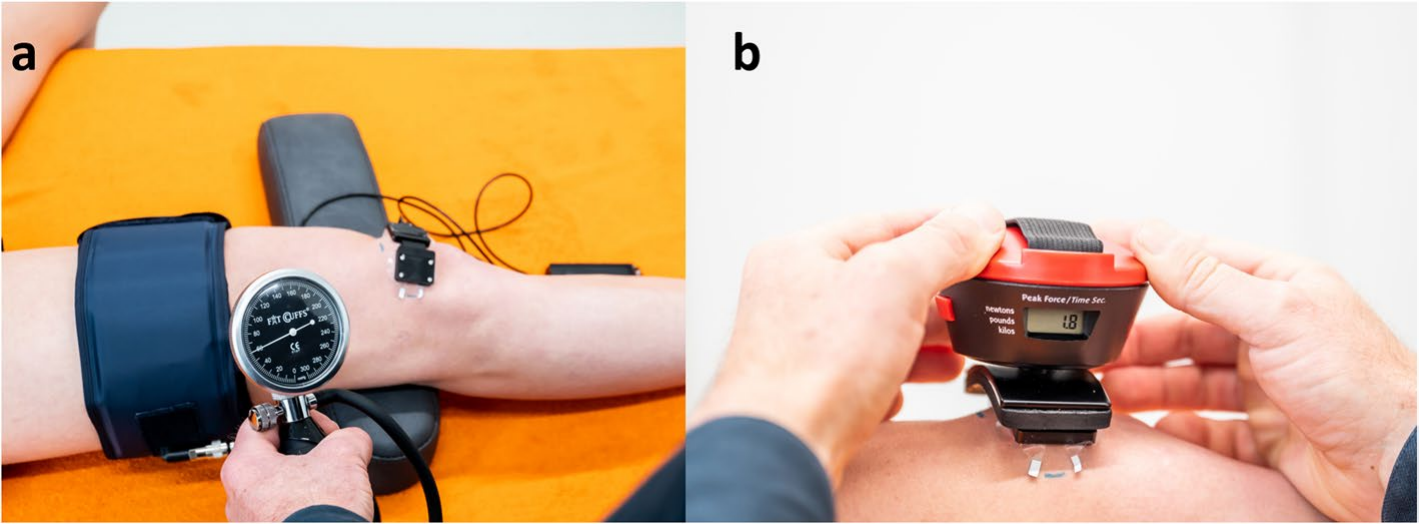
**a** Experiment 1 ‘Venous Occlusion’ and 3b Experiment 2 ‘Skin Compression’. (Note: Absence of the opaque cloth to visualise the set-up. During measurements the sensor was covered)

As disturbed venous outflow of the patella is a key feature of the homeostasis model [2], an experiment assessed changes of the NIRS signal under venous occlusion of the dominant leg. Using a thigh cuff (Leg Cuff V3.1; Fitcuffs, Odder, Denmark) positioned 10 cm proximal to the mark at the patella’s proximal edge, venous occlusion was achieved. Before the start of the experiment, participants lay in supine position for 15 min with the knee flexed in 20 degrees. Examiner AW set this angle with an extendable goniometer (Lafayette, IN, US). Examiner AW visually inspected the NIRS signal for stability and the 3-min baseline was started once stability was confirmed. Subsequently, the cuff was inflated to 60 mmHG for one minute, inducing venous occlusion without disturbing arterial blood flow (Fig. 3a) [21, 35].

#### Experiment 2 ‘Skin Compression’

Based on the previously described light-detector distance of the NIRS optode, the patellar bone is the main target tissue of the NIRS measurement. By creating skin compression, blood flow of the skin is compromised, but the NIRS signal is expected not to change significantly. Skin compression was achieved with a handheld dynamometer (Biofet; Mustec BV, Almere, The Netherlands). When the NIRS signal was stable (visual inspection), the baseline of Experiment 2 ‘Skin Compression’ was started three minutes after finishing Experiment 1 ‘Venous Occlusion’. After the 3-min baseline the handheld dynamometer was placed on the NIRS sensor to create mechanical skin compression of 100 mmHG (135 gr/cm^2^ or 1.8 kg) for one minute (Fig. 3b) [35]. After finishing both experiments, NIRS measurements were continued for 15 s (recovery time).

### Test–Retest reliability

The test–retest reliability of NIRS in the patellar bone was evaluated for two clinically relevant activities for PFP patients: prolonged sitting with the knees flexed and stair descent. Despite the absence of active loading of the patellofemoral joint, more than 80% of PFP patients experience pain with prolonged sitting [10]. Previous research recommends to explore the mechanism of sitting pain [10]. Additionally, stair climbing is among the most provoking activities of daily living for PFP patients [6]. To evaluate the test–retest reliability five PFP patients and 15 healthy controls performed both clinically relevant activities twice with three to five days in between.

#### Activity 1 ‘Prolonged Sitting’

**Fig. 4:**
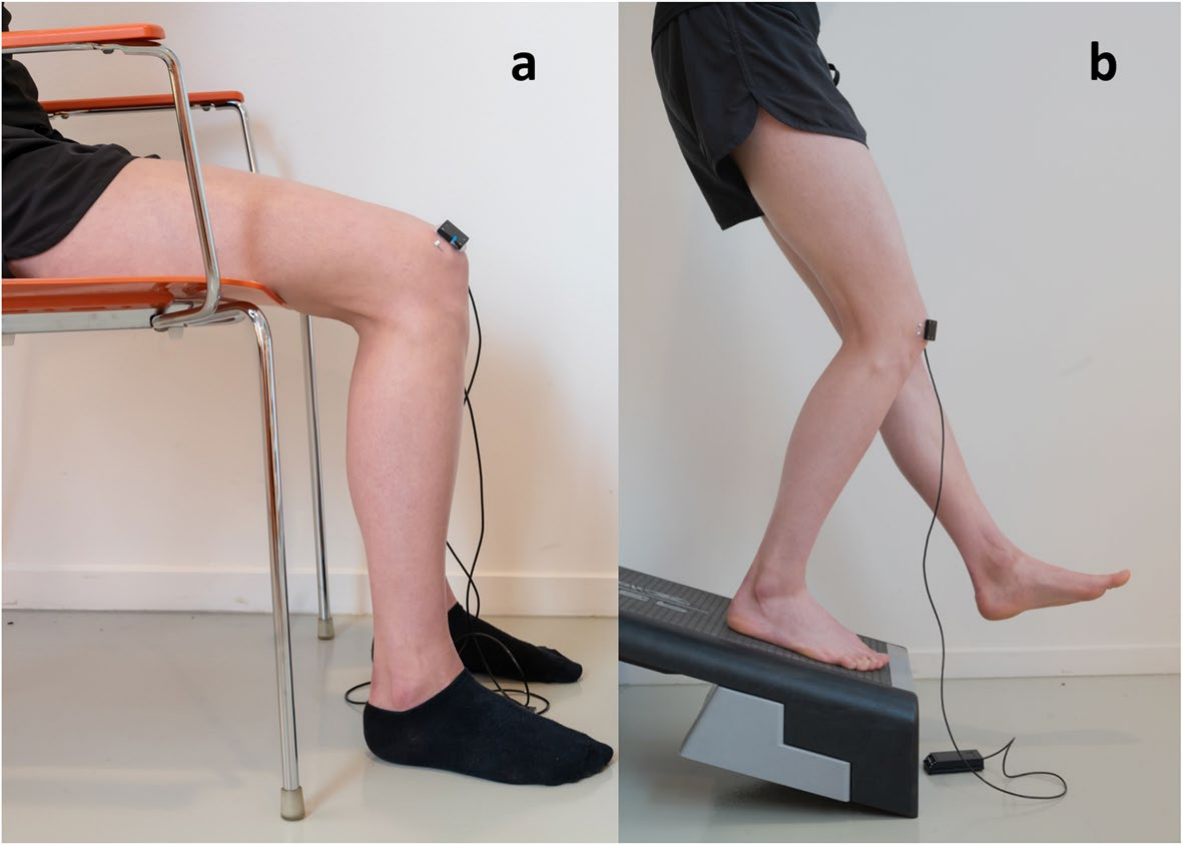
**a** Activity 1 ‘Prolonged Sitting’ and 4b Activity 2 ‘Stair Descent´. (Note: Absence of the opaque cloth to visualise the set-up, during measurements the sensor was covered)

After a 15-min rest period with the participants’ knees flexed at 20 degrees and stable NIRS signal (visual inspection), a 3-min baseline was established in the same position. Subsequently, knees were flexed to 90 degrees for 30 min (Fig. 4a), followed by returning to 20 degrees of flexion for five minutes. Knee angles were verified using an extendable goniometer (Lafayette, IN, US).

#### Activity 2 ‘Stair Descent’

Stair descent was simulated using the previously developed Decline Step-Down Test (DSDT) [37]. Following a 15-min rest in a standing position and ensuring stable NIRS signal (visual inspection), a 3-min baseline was established while standing on the decline step-down set-up with extended knees. Participants performed a motion simulating stair descent (45 degrees of knee flexion) and held this for one minute (Fig. 4b). Correctness of this knee angle was confirmed with an extendable goniometer (Lafayette, IN, US). After a 3-min rest, the procedure was repeated for the other leg, with the starting leg randomised resulting in half of the participants starting with the left and the other half with the right leg.

### Blinding

Blinding for health status (PFP patient or healthy control) was assured. Examiner MO was responsible for participant inclusion and exclusion, while examiner AW conducted measurements without knowledge of health status.

### Sample size

Given the absence of information regarding expected effect sizes in NIRS studying patellar bone hemodynamics, the sample size was maximised based on available resources. A convenient sample of five PFP patients (5–10 symptomatic knees) and 15 healthy participants (30 knees) was anticipated.

### Statistical analysis

Normality of distribution was assessed using visual inspection and the Shapiro–Wilk test. The participants’ demographic data were described using means (*M*), standard deviations (*SD*) for continuous variables, and percentages (%) for dichotomous variables. Differences in continuous baseline characteristics between PFP patients and healthy controls were analysed using Student’s *t*-test for normally distributed data or Mann–Whitney *U*-test for not normally distributed data. Differences in categorical baseline characteristics were analysed using Fisher’s exact test.

For both experiments, mean and standard deviation for changes in HHb and O_2_Hb between baseline and experimental measurement (ΔBas_Exp1 and ΔBas_Exp2) were calculated separately for each of the three optodes (30, 35, 40 mm). The first and last three seconds were removed to eliminate any influence of movement artifacts. Additionally, concentrations of HHb and O_2_Hb on the three optodes were visualised with plots.

Effect size (*ES*) was calculated using Cohen’s *d* to present the magnitude of the change between baseline and experimental measurement. An *ES* of 0.2 was considered small, 0.5 medium, and ≥ 0.8 large [7].

A NIRS sensor was placed on both knees of each participant. For the statistical analysis, the knees were included separately. For PFP patients, only the symptomatic knees were included, excluding non-symptomatic knees from the statistical analysis. Both knees of healthy controls were included.

Test–retest reliability of NIRS in the patellar bone was assessed for both activities. For Activity 1 ‘Prolonged Sitting’ four changes in HHb and O_2_Hb were assessed: 1) change between baseline and first 10 min of sitting with knees flexed (ΔBas_Sit0-10), 2) change between baseline and second 10 min of sitting with knees flexed (ΔBas_Sit10-20), 3) change between baseline and third 10 min of sitting with knees flexed (ΔBas_Sit20-30), 4) change between third 10 min of sitting with knees flexed and five minutes of recovery time (ΔSit20-30_Rec). For Activity 2 ‘Stair Descent’, the change in HHb and O_2_Hb between baseline and 45 degrees of knee flexion was assessed (ΔBas_Step).

Changes were calculated for each concentration (HHb, O_2_Hb) and optode separately (30, 35, 40 mm). Test–retest reliability was assessed using intraclass correlation coefficient (*ICC*), standard error of the measurement (*SEM*), and the smallest detectable change (*SDC*) for each change in HHb and O_2_Hb. Moreover, the *ICC*, *SEM* and *SDC* were determined for PFP patients and healthy controls separately. *ICC*_*2,1*_ was calculated using a single-rating, absolute agreement, two-way mixed effect model, with values indicating slight agreement (0.0 to 0.2), fair agreement (0.21 to 0.40), moderate agreement (0.41 to 0.60), substantial agreement (0.61 to 0.80), and almost perfect agreement (0.81 to 1.0) [28]. Measurement error as an indicator of the measurement's precision was assessed by the standard error of measurement (*SEM*) and calculated according to the formula: *SD* x√(1-*ICC*), *SD* being the standard deviation from mean scores and ICC for test–retest reliability [14]. The smallest detectable change (*SDC*) was calculated at group level, according to the formula: *SEM* × 1.96√2/√*n* [53].

NIRS data collection was performed with Oxysoft, version 3.2.72 (Artinis, Elst, The Netherlands). The Oxysoft files were converted into MATLAB files (R2020a version 9.8.0.1323502, Mathworks, Natick, USA). MATLAB was used to filter the raw values with a moving average filter, and calculate changes between baseline and experimental measurements. The outcomes were entered in Excel (Microsoft Office version 16), and the statistical analyses were performed using SPSS version 28.0 with a level of significance set at *p* < 0.05.

## Results

**Fig. 5:**
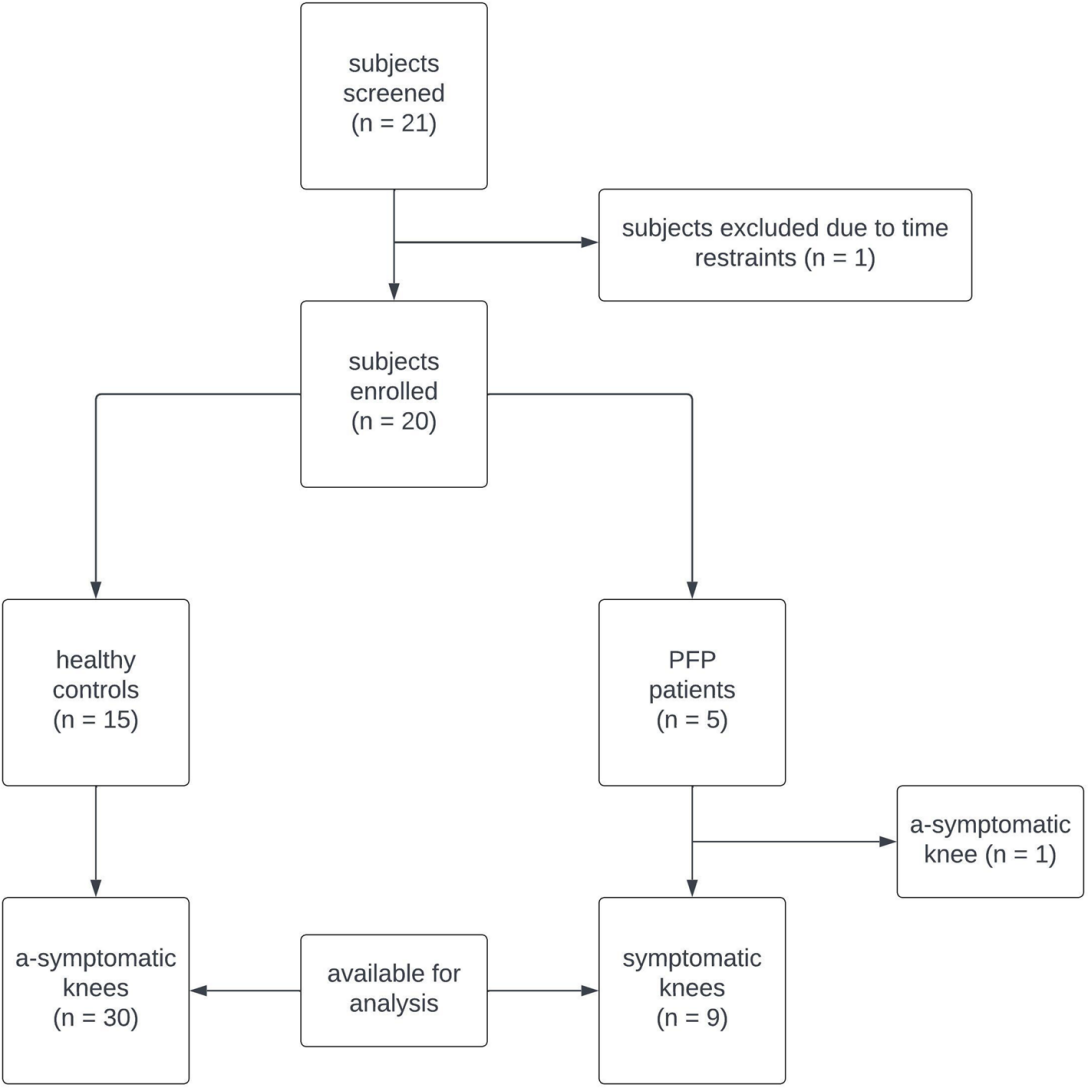
Flowchart of the inclusion process

Out of 21 subjects screened for participation, 20 were included, and one was excluded due to personal time constraints (Fig. 5).

Table 2 describes baseline characteristics of all participants. Fifteen healthy controls (10 females [66.7%], mean age 21.0 years [*SD* = 2.6] and mean BMI 22.0 kg/m^2^ [*SD* = 2.7]) and five patients with PFP (4 females [80.0%], mean age 22.2 years [*SD* = 2.6] and mean BMI 23.8 kg/m^2^ [*SD* = 3.1]) were included. Patients with PFP differed only in the AKPS score compared to healthy controls. The mean AKPS score of PFP patients (*M* = 87.2; *SD* = 3.8) was significantly lower compared to healthy controls (*M* = 98.3; *SD* = 2.1), (*t*(18) = 8.81; *p* < 0.001, *d* = 3.62), indicating correct group allocation.

**Table 2:** Baseline characteristics

	**Experiments**	**Reliability**	
	**Controls**	**Controls**	**PFP patients**
**Participants, *n***	10	15	5
**Female, *n* (%)**	6 (60.0)	10 (66.7)	4 (80.0)
**Age (yrs)**	21.7 (2.7)	21.0 (2.6)	22.2 (3.8)
**BMI (kg/m**^**2**^**)**	22.2 (2.1)	22.0 (2.7)	23.8 (3.1)
**Bilateral PFP, *n* (%)**	n/a	n/a	4 (80.0)
**Symptom duration (mos)**	n/a	n/a	47 (35.9)
**VAS-W (0–10)**	n/a	n/a	5 (1.1)
**Smoking, *n* (%)**	0 (0.0)	0 (0.0)	2 (40.0)
**Blood pressure (mmHg)**			
Systolic	120.8 (11.1)	120.7 (9.1)	124.1 (8.0)
Diastolic	80.7 (9.3)	80.6 (8.6)	80.7 (10.3)
**Sport participation (hrs/wk)**	7.5 (8.4)	6.9 (8.4)	4.4 (1.5)
**Tegner (0–10)**	6.5 (1.0)	6.4 (0.9)	6.2 (1.8)
**AKPS (0–100)**	98.4 (2.5)	98.3 (2.1) ***	87.2 (3.8) ***
**Patella (mm)**			
Width	51.4 (3.1)	51.2 (3.2)	50.1 (3.2)
Skinfold	7.0 (1.2)	7.1 (1.8)	7.7 (1.5)

Data are numbers (percentages), mean (standard deviation) or median (interquartile range 25%-75%)

*Abbreviations: n* number, yrs years, BMI body mass index in kilogram bodyweight per m^2^, *mos* months, *VAS-W* visual analogue scale for worst pain, *mmHg*, millimetre of mercury, *hrs/wk* hours per week, *AKPS* anterior knee pain scale, *mm* millimetre, *n/a* not applicable

^***^, *p*-value < .001

### Experiments

**Fig. 6:**
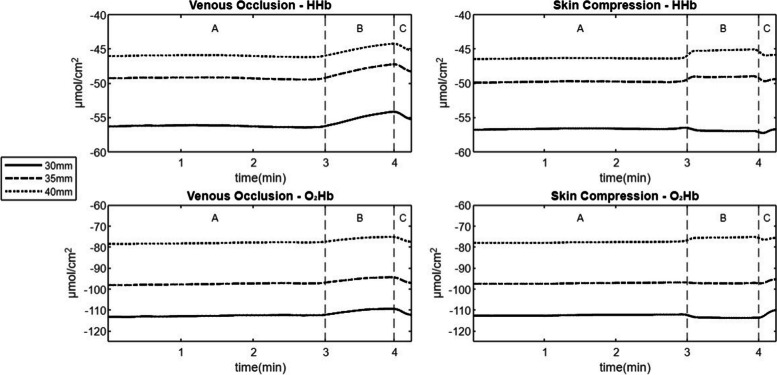
Plotted means of HHb and O_2_Hb (in µmol/cm.^2^) of Experiment 1 ‘Venous Occlusion’ and Experiment 2 ‘Skin Compression’. *Abbreviations:* HHb, deoxygenated hemoglobine; O_2_Hb, oxygenated hemoglobine for each optode (30, 35, 40 mm); min, minutes; A, baseline (3 min); B, experimental measurement (venous occlusion / skin compression) (1 min); C, recovery time (15 s)

Both experiments were conducted on 10 consecutively enrolled healthy participants before their performance of Activity 1 ‘Prolonged Sitting’ and Activity 2 ‘Stair Descent’. All participants completed both activities during the two visits as planned. Table 3 presents the mean and standard deviation for the change in HHb and O_2_Hb between baseline and experimental measurements. Significant increases in both HHb and O_2_Hb concentrations were observed on all optodes (30, 35, 40 mm) during Experiment 1 ‘Venous Occlusion’ (*p* < 0.001 – 0.002). Additionally, statistically significant increases in HHb and O_2_Hb were noted only on the 40 mm optode during Experiment 2 ‘Skin Compression’ (*p* = 0.004 – 0.006). No statistically significant changes (*p* = 0.075 – 0.61) were observed on the 30 mm and 35 mm optodes during Experiment 2 ‘Skin Compression’. Plots of HHb and O_2_Hb during both experiments are presented in Fig. 6.

**Table 3:** Changes in HHb and O_2_Hb for Experiment 1 ‘Venous Occlusion’ and Experiment 2 ‘Skin Compression’

***n*= 10**	**30 mm optode**		**35 mm optode**		**40 mm optode**	
**HHb**	Mean (*SD*)	*p*-value *ES*	Mean (*SD*)	*p*-value *ES*	Mean (*SD*)	*p*-value *ES*
ΔBas_Exp1^a^	1.1 (0.7)	*p* < .001; *d* = 0.22***	1.2 (0.8)	*p* < .001; *d* = 0.24 ***	1.0 (0.5)	*p* < .001; *d* = 0.21***
ΔBas_Exp2^b^	-0.3 (1.5)	*p* = .61	0.9 (1.3)	*p* = .075	1.1 (1.0)	*p* = 0.006; *d* = 0.27**
**O**_**2**_**Hb**						
ΔBas_Exp1^a^	2.0 (1.3)	*p* < .001; *d* = 0.34 ***	2.0 (1.5)	*p* = .002; *d* = 0.37**	1.9 (0.9)	*p* < . 001; *d* = 0.35***
ΔBas_Exp2^b^	-0.9 (2.2)	*p* = .25	0.4 (2.2)	*p* = .55	2.0 (1.6)	*p* = .004; *d* = 0.37**

*Abbreviations: HHb* deoxygenated hemoglobine, *O*_*2*_*Hb* oxygenated hemoglobine, *SD* standard deviation, *ES* effect size (Cohens ‘*d*’)

^**^ and ***, *p*-value < .01 and < .001, respectively

^a^The change (in µmol/cm^2^) between the baseline and experiment 1 ‘Cuff Occlusion

^b^The change (in µmol/cm^2^) between the baseline and experiment 2 ‘Skin Compression

### Test–retest reliability of Activity 1 ‘Prolonged Sitting’

Table 4 shows results of reliability analysis for HHb for each optode (30, 35, 40 mm). Overall, *ICCs* for the 30 mm optode indicated moderate to substantial agreement for total (n = 39), healthy control (*n* = 30) and patient sample (*n* = 9). However, *ICCs* for the 35 and 40 mm optode showed moderate to substantial agreement for total and healthy control sample but slight to fair agreement in the patient sample.

**Table 4:** Test–retest reliability of Activity 1 ‘Prolonged Sitting’ (HHb)

	**30 mm optode**			**35 mm optode**			**40 mm optode**		
**Total** **(*n* = 39)**	*ICC* (*95% CI*)	*SEM*	*SDC*	*ICC* (*95% CI*)	*SEM*	*SDC*	*ICC* (*95% CI*)	*SEM*	*SDC*
ΔBas_Sit0-10^a^	0.63 (0.40, 0.79)	1.1	3.1	0.50 (0.22, 0.70)	1.2	3.3	0.56 (0.30, 0.74)	1.0	2.7
ΔBas_Sit10-20^b^	0.69 (0.48, 0.83)	1.1	3.1	0.56 (0.31, 0.74)	1.2	3.3	0.63 (0.39, 0.79)	1.0	2.7
ΔBas_Sit20-30^c^	0.71 (0.51, 0.84)	1.1	3.1	0.60 (0.36, 0.77)	1.2	3.3	0.67 (0.46, 0.82)	1.0	2.7
ΔSit20-30_Rec^d^	0.70 (0.49, 0.83)	0.7	2.0	0.65 (0.43, 0.80)	0.7	2.1	0.61 (0.37, 0.78)	0.8	2.1
**Controls** **(*n* = 30)**									
ΔBas_Sit0-10^a^	0.63 (0.36, 0.81)	1.2	3.2	0.50 (0.19, 0.73)	1.2	3.4	0.58 (0.29, 0.78)	1.0	2.8
ΔBas_Sit10-20^b^	0.70 (0.46, 0.85)	1.2	3.2	0.58 (0.28, 0.77)	1.2	3.4	0.66 (0.39, 0.82)	1.0	2.8
ΔBas_Sit20-30^c^	0.72 (0.49, 0.86)	1.2	3.2	0.61 (0.33, 0.79)	1.2	3.4	0.70 (0.46, 0.85)	1.0	2.8
ΔSit20-30_Rec^d^	0.75 (0.53, 0.87)	0.7	1.8	0.69 (0.45, 0.84)	0.8	2.1	0.68 (0.42, 0.83)	0.7	2.0
**PFP** **(*n* = 9)**									
ΔBas_Sit0-10^a^	0.51 (-0.09, 0.86)	0.9	2.4	0.16 (-0.47, 0.71)	0.9	2.4	0.05 (-0.50, 0.64)	0.7	2.0
ΔBas_Sit10-20^b^	0.50 (-0.11, 0.85)	0.9	2.5	0.14 (-0.44, 0.69)	0.9	2.4	0.10 (-0.40, 0.65)	0.8	2.1
ΔBas_Sit20-30^c^	0.47 (-0.15, 0.85)	0.9	2.5	0.12 (-0.46, 0.68)	0.9	2.4	0.06 (-0.43, 0.63)	0.8	2.1
ΔSit20-30_Rec^d^	0.55 (-0.03, 0.87)	0.8	2.3	0.41 (-0.18, 0.82)	0.7	1.8	0.22 (-0.26, 0.71)	0.7	1.9

*Abbreviations: HHb* deoxygenated hemoglobine, *ICC* intraclass correlation coefficient, *CI* confidence interval, *SEM* standard error of measurement (in µmol/cm^2^), *SDC* smallest detectable change (in µmol/cm^2^)

^a^Change between baseline and first 10 min of sitting with the knees flexed

^b^Change between baseline and second 10 min of sitting with the knees flexed

^c^Change between baseline and third 10 min of sitting with the knees flexed

^d^Change between third 10 min of sitting with the knees flexed and 5 min of recovery time

For O_2_Hb (Table 5), *ICCs* for the 30 mm and 40 mm optodes indicated moderate to almost perfect agreement for total, healthy control and patient sample. However, *ICCs* for the 35 mm optode showed moderate to substantial agreement for total and patient sample but fair agreement in the healthy control sample for the change between baseline and second 10 min of sitting with knees flexed.

**Table 5:** Test–retest reliability of Activity 1 ‘Prolonged Sitting’ (O_2_Hb)

	**30 mm optode**			**35 mm optode**			**40 mm optode**		
**Total** **(*n* = 39)**	*ICC* (*95% CI*)	*SEM*	*SDC*	*ICC* (*95% CI*)	*SEM*	*SDC*	*ICC* (*95% CI*)	*SEM*	*SDC*
ΔBas_Sit0-10^a^	0.80 (0.66, 0.89)	1.4	0.6	0.68 (0.47, 0.82)	1.4	0.6	0.68 (0.47, 0.82)	1.1	0.5
ΔBas_Sit10-20^b^	0.73 (0.54, 0.85)	1.6	0.7	0.60 (0.35, 0.77)	1.6	0.7	0.54 (0.27, 0.73)	1.3	0.6
ΔBas_Sit20-30^c^	0.77 (0.60, 0.87)	1.5	0.7	0.62 (0.38, 0.78)	1.6	0.7	0.59 (0.34, 0.76)	1.3	0.6
ΔSit20-30_Rec^d^	0.86 (0.75, 0.92)	1.8	0.8	0.85 (0.73, 0.92)	1.6	0.7	0.80 (0.66, 0.89)	1.3	0.6
**Controls** **(*n* = 30)**									
ΔBas_Sit0-10^a^	0.68 (0.43, 0.84)	1.4	0.7	0.51 (0.19, 0.73)	1.5	0.7	0.61 (0.33, 0.80)	1.1	0.6
ΔBas_Sit10-20^b^	0.56 (0.25, 0.76)	1.8	0.9	0.39 (0.03, 0.66)	1.7	0.9	0.43 (0.08, 0.68)	1.4	0.7
ΔBas_Sit20-30^c^	0.66 (0.39, 0.82)	1.7	0.9	0.49 (0.16, 0.72)	1.7	0.9	0.53 (0.21, 0.75)	1.4	0.7
ΔSit20-30_Rec^d^	0.80 (0.62, 0.90)	1.9	1.0	0.78 (0.58, 0.89)	1.7	0.8	0.73 (0.51, 0.86)	1.3	0.7
**PFP** **(*n* = 9)**									
ΔBas_Sit0-10^a^	0.92 (0.71, 0.98)	1.3	1.2	0.88 (0.57, 0.97)	1.3	1.2	0.83 (0.41, 0.96)	1.0	0.9
ΔBas_Sit10-20^b^	0.94 (0.76, 0.99)	1.2	1.1	0.90 (0.62, 0.98)	1.1	1.0	0.82 (0.39, 0.96)	1.0	0.9
ΔBas_Sit20-30^c^	0.95 (0.80, 0.99)	1.0	0.9	0.89 (0.60, 0.98)	1.0	1.0	0.80 (0.34, 0.95)	0.9	0.9
ΔSit20-30_Rec^d^	0.95 (0.80, 0.99)	1.6	1.5	0.96 (0.84, 0.99)	1.1	1.0	0.93 (0.72, 0.98)	1.0	0.9

*Abbreviations: O*_*2*_*Hb* oxygenated hemoglobine, *ICC* intraclass correlation coefficient, *CI* confidence interval, *SEM* standard error of measurement (in µmol/cm^2^), *SDC* smallest detectable change (in µmol/cm^2^)

^a^Change between baseline and first 10 min of sitting with the knees flexed

^b^Change between baseline and second 10 min of sitting with the knees flexed

^c^Change between baseline and third 10 min of sitting with the knees flexed

^d^Change between third 10 min sitting with the knees flexed and 5 min of recovery time

### Test–retest reliability of Activity 2 ‘Stair Descent’

Table 6 presents results of reliability analysis for HHb for each optode (30, 35, 40 mm). *ICCs* for the 35 mm optode showed moderate to substantial agreement for total, healthy control and patient sample. However, *ICCs* for the 30 mm and 40 mm optode showed substantial agreement for total and healthy control sample but slight to fair agreement in the patient sample.

**Table 6:** Test–retest reliability of Activity 2 ‘Stair Descent’ (HHb)

	**30 mm optode**			**35 mm optode**			**40 mm optode**		
**Total** **(*n* = 39)**	*ICC* (*95% CI*)	*SEM*	*SDC*	*ICC* (*95% CI*)	*SEM*	*SDC*	*ICC* (*95% CI*)	*SEM*	*SDC*
ΔBas_Step^a^	0.68 (0.47, 0.82)	1.7	0.7	0.65 (0.41, 0.80)	1.1	0.5	0.62 (0.38, 0.78)	0.9	0.4
**Controls** **(*n* = 30)**									
ΔBas_Step^a^	0.72 (0.49, 0.86)	1.7	0.9	0.68 (0.42, 0.83)	1.1	0.5	0.73 (0.50, 0.86)	0.8	0.4
**PFP** **(*n* = 9)**									
ΔBas_Step^a^	0.34 (-0.46, 0.81)	1.6	1.4	0.50 (-0.18, 0.86)	1.1	1.0	0.16 (-0.42, 0.70)	1.1	1.1

*Abbreviations: HHb* deoxygenated hemoglobine, *ICC* intraclass correlation coefficient, *CI* confidence interval, *SEM* standard error of measurement (in µmol/cm^2^), *SDC* smallest detectable change (in µmol/cm^2^)

^a^Change between baseline and 45 degrees of knee flexion

For O_2_Hb (Table 7), *ICCs* for the 35 mm optode showed moderate to substantial agreement for total, healthy control and patient sample. However, *ICCs* for the 30 mm and 40 mm optode showed moderate agreement for total and healthy control sample but slight to fair agreement in the patient sample.

**Table 7:** Test–retest reliability of Activity 2 ‘Stair Descent’ (O_2_Hb)

	**30 mm optode**			**35 mm optode**			**40 mm optode**		
**Total** **(*n* = 39)**	*ICC* (*95% CI*)	*SEM*	*SDC*	*ICC* (*95% CI*)	*SEM*	*SDC*	*ICC* (*95% CI*)	*SEM*	*SDC*
ΔBas_Step^a^	0.45 (0.17, 0.67)	2.7	1.2	0.54 (0.27, 0.73)	1.9	0.8	0.42 (0.12, 0.65)	1.8	0.8
**Controls** **(*n* = 30)**									
ΔBas_Step^a^	0.48 (0.16, 0.71)	2.6	1.3	0.51 (0.18, 0.73)	1.9	0.9	0.55 (0.24, 0.76)	1.5	0.8
**PFP** **(*n* = 9)**									
ΔBas_Step^a^	0.39 (-0.38, 0.82)	2.8	2.5	0.62 (-0.03, 0.90)	2.0	1.8	0.08 (-0.61, 0.68)	2.4	2.2

*Abbreviations: O*_*2*_*Hb* oxygenated hemoglobine, *ICC* intraclass correlation coefficient, *CI* confidence interval, *SEM* standard error of measurement (in µmol/cm^2^), *SDC* smallest detectable change (in µmol/cm^2^)

^a^Change between baseline and 45 degrees of knee flexion

## Discussion

This study, for the first time, investigated characteristics of the NIRS signal during experiments affecting hemodynamics of the patella in healthy controls and to evaluate test–retest-reliability in both PFP patients and healthy controls. The main findings revealed that 1) venous occlusion significantly increased the NIRS signal (HHb and O_2_Hb) derived from the patella, while no significant changes occurred during skin compression, and 2) the test–retest reliability of NIRS measures (HHb and O_2_Hb) derived from the patella showed moderate to almost perfect agreement in positions clinically relevant for PFP patients. These findings indicate that while NIRS measurements of the patella are sufficiently reliable as research application to compare real-time bone hemodynamics in PFP patients and healthy controls, they might not be suitable for clinical application for individual subject evaluation.

Venous occlusion of the thigh resulted in an increase of the NIRS signal (HHb and O_2_Hb) on all optodes (30, 35, 40 mm). With venous occlusion, there is (arterial) inflow while (venous) outflow is compromised, which results in increased concentrations of HHb and O_2_Hb. This is in line with previous measurements with this device [3] and other studies assessing blood flow in the forearm with NIRS and strain-gauge plethysmography [13, 34]. Näslund et al. [35] found reduced intraosseous blood flow of the patella using photoplethysmography during venous occlusion. Experiment 1 ‘Venous Occlusion’ mimicked vascular insufficiency and observed NIRS signal changes could be expected when comparing bone hemodynamics of the patella in PFP patients and healthy controls.

The NIRS signal (HHb and O_2_Hb) did not increase on the 30 mm and 35 mm optode when skin compression of the patella was applied. This is in line with our hypothesis, since the measurement depth of this NIRS device (15 mm to 20 mm) is deeper than the thickness of the skin. The observed mean prepatellar skinfold thickness was 7.7 mm and 7.1 mm in PFP patients and healthy controls, resulting in skin thickness (skinfold dividing by two) of 3.9 mm and 3.6 mm, respectively. A skin thickness of less than 4 mm has a non-significant confounding contribution on the NIRS signal [18]. Surprisingly, a statistically significant (*p* = 0.004 – 0.006) increase of the NIRS signal was found on the 40 mm optode. More research is needed on this specific topic.

Furthermore, the patella width in the current study was between 50.1 mm (patient sample) and 51.4 (total sample). Porteus et al. [38] reported a ‘Normal Index of Patella Width: Thickness’ of 1.8:1, which translates into an estimated patella thickness of 27.8 mm to 28.6 mm including cartilage. We conclude that even the 40 mm optode (measurement depth of 20 mm) still measures in the patellar bone.

The *ICCs* of the 30 mm optode (0.47 – 0.95) showed moderate to almost perfect agreement during Activity 1 ‘Prolonged Sitting’ and therefore this optode is most suitable to compare changes of HHb and O_2_Hb concentrations in PFP patients and healthy controls. The *ICCs* of the 35 mm optode (0.50 – 0.68) showed moderate to substantial agreement during Activity 2 ‘Stair Descent’ and therefore this is most suitable to compare changes of HHb and O_2_Hb concentrations in PFP patients and healthy controls. Notably, in PFP patients during Activity 1 ‘Prolonged Sitting” the *ICCs* for HHb of the 35 mm and 40 mm optode indicated slight to fair agreement only, while the *ICCs* for O_2_Hb indicated almost perfect agreement on all optodes. Given the large 95% confidence intervals this observed difference may be due to the small sample size (*n* = 9). This also applies to the fair reliability of the 30 mm and 40 mm optodes found during Activity 2 ‘Stair Descent’ in PFP patients.

Generally, this study found wide variability of measures of reliability for both Activity 1 ‘Prolonged Sitting’ and Activity 2 ‘Stair Descent’. Based on results of the current study, it is unclear what is attributable to normal biological variation of hemoglobine concentrations in bone tissue or to measurement error. Regarding biological variability, heterogeneity of bone tissue and lower metabolic rate than muscle tissue, including lower sampling volume of HHb and O_2_Hb in bone, are known barriers in vascular measurement of bone [32].

Regarding measurement error, a random source of bias could be introduced by the determination of degrees of knee flexion during NIRS measurements by goniometer assessments. Measurement errors up to 10 degrees have been reported [31]. Generally, there are no known optode-specific sources of bias and all optodes measure changes of HHb and O_2_Hb concentrations at a tissue depth of 15—20 mm [3]. All other known sources of bias like no exercising 24 h before and rest time prior to measurements, and standardisation of optode placement have been taken into account. Due to this observed wide variability of measures of reliability, NIRS cannot play a role in the evaluation of patellar blood flow in the clinical setting on an individual level, but is limited to the cautious application in scientific research to evaluate differences in patellar blood flow on a group level.

Other clinical studies on blood flow of the anterior knee in PFP patients examined skin temperature by thermal imaging (only superficially) [46], pulsatile blood flow by photoplethysmography (only for five minutes) [36], or blood perfusion by dynamic contrast-enhanced MRI (only in knee extension) [51]. Our study is the first to study real-time bone hemodynamics of the patella continuously and in clinically relevant positions for PFP patients. We suggest that this protocol also can be used to study hemodynamics of the patella in patients with other anterior knee pain conditions, e.g., patellofemoral osteoarthritis. Whether the clinimetric properties of the assessment are similar should be subject of further study.

The current study is not without limitations. As mentioned before, the number of test–retest observations of the patient sample is considered to be low compared to the number of observations of the healthy control sample. The low number of patient observations affects statistical power and increases confidence intervals of the *ICCs*. Unfortunately, even if effect sizes would have been known prior to the start of the current study, given the limited resources of our study group, it would not have been possible to increase the number of participating PFP patients. It is recommended to collect additional data on the reproducibility of this NIRS protocol in PFP patients.

Another limitation was the absence of randomisation of the order of the experiments. During preparation of the study, the NIRS signal required 10 min to normalise after skin compression while the same signal normalised within one minute after venous occlusion. To avoid unnecessary burden for the participants, we therefore decided to perform the experiments without randomisation.

The performance of Activity 2 `Stair Descent` mimics stair descent in the real world only in a limited way, since stair descent is much more dynamic, but also much more random, resulting in movement variability. Unfortunately, movement variability would introduce major artefacts into the NIRS measurement. Nevertheless, the chosen performance here creates patellofemoral joint loading in combination with active muscle contraction.

## Conclusions

The current study found NIRS measurements of the patella to be affected by venous occlusion of the thigh, but not by skin compression. Furthermore, NIRS measurements seem sufficiently reliable as research application to compare real-time patellar bone hemodynamics in PFP patients and healthy controls. This opens a window of opportunity to determine differences in hemodynamics of the patella between patients and healthy controls and may help to improve effectiveness of evidence-based treatment strategies for PFP patients.

## Data Availability

Research data will be available upon reasonable request.

## Abbreviations

AKPS: Anterior Knee Pain Scale
BMI: Body Mass Index
CI: Confidence Interval
DPF: Differential Pathlength Factor
DSDT: Decline Step-Down Test
ES: Effect Size
HHb: Deoxygenated Hemoglobine
ICC: Intraclass Correlation Coefficient
ISAK: International Society for the Advancement of Kinanthropometry
LLROM: Lower Limb Range Of Motion
NIRS: Near-Infrared Spectroscopy
O_2_Hb: Oxygenated Hemoglobine
PFM: Patellofemoral Maltracking
PFP: Patellofemoral Pain
SD: Standard Deviation
SDC: Smallest Detectable Change
SEM: Standard Error of the Measurement
VAS: Visual Analogue Scale
